# Effect of Embryo Banking on U.S. National Assisted Reproductive Technology Live Birth Rates

**DOI:** 10.1371/journal.pone.0154620

**Published:** 2016-05-09

**Authors:** Vitaly A. Kushnir, David H. Barad, David F. Albertini, Sarah K. Darmon, Norbert Gleicher

**Affiliations:** 1 The Center for Human Reproduction, New York, NY, United States of America; 2 Wake Forest School of Medicine, Winston-Salem, NC, United States of America; 3 Foundation for Reproductive Medicine, New York, NY, United States of America; 4 University of Kansas Medical Center, Kansas City, KS, United States of America; 5 The Rockefeller University, New York, NY, United States of America; Peiking University Third Hospital, CHINA

## Abstract

**Background:**

Assisted Reproductive Technology (ART) reports generated by the Centers for Disease Control and Prevention (CDC) exclude embryo banking cycles from outcome calculations.

**Methods:**

We examined data reported to the CDC in 2013 for the impact of embryo banking exclusion on national ART outcomes by recalculating autologous oocyte ART live birth rates. Inflation of reported fresh ART cycle live birth rates was assessed for all age groups of infertile women as the difference between fresh cycle live births with reference to number of initiated fresh cycles (excluding embryo banking cycles), as typically reported by the CDC, and fresh cycle live births with reference to total initiated fresh ART cycles (including embryo banking cycles).

**Results:**

During 2013, out of 121,351 fresh non-donor ART cycles 27,564 (22.7%) involved embryo banking. The proportion of banking cycles increased with female age from 15.5% in women <35 years to 56.5% in women >44 years. Concomitantly, the proportion of thawed cycles decreased with advancing female age (P <0.0001). Exclusion of embryo banking cycles led to inflation of live birth rates in fresh ART cycles, increasing in size in parallel to advancing female age and utilization of embryo banking, reaching 56.3% in women age >44. The inflation of live birth rates in thawed cycles could not be calculated from the publically available CDC data but appears to be even greater.

**Conclusions:**

Utilization of embryo banking increased during 2013 with advancing female age, suggesting a potential age selection bias. Exclusion of embryo banking cycles from national ART outcome reports significantly inflated national ART success rates, especially among older women.

**Précis:**

Exclusion of embryo banking cycles from US National Assisted Reproductive Technology outcome reports significantly inflates reported success rates especially in older women.

## Introduction

The US National Assisted Reproductive Technology (ART) Surveillance System (NASS), under the Fertility Clinic Success Rate and Certification Act (FCSRCA) administered by the Centers for Disease Control and Prevention (CDC), was intended to give the public access to transparent and understandable ART pregnancy success rates in reference to number of ovarian stimulation procedures attempted. [1,2] Since this legislation was passed, ART in the U.S. has undergone considerable changes. One rapidly growing practice change, cryopreservation of entire embryo cohorts (embryo banking), is intended to lead to improved treatment outcomes in subsequent thawed embryo transfer cycles. [3] This practice is often accompanied by accumulation of embryos from multiple fresh cycles. Another recent addition to ART, preimplantation genetic screening (PGS) at blastocyst stage, also often involves cryopreservation of all embryos without immediate transfer. [4] These modifications of ART practice resulted in significant loss of desired transparency of national ART reporting since embryo banking made it difficult to track ART cycle outcomes reliably.

The main reason for this loss of transparency was the CDC’s decision to allow exclusion of embryo banking cycles, from success rate calculations because no immediate pregnancy outcome is achievable if no embryo transfer occurs in the same ART cycle. The purpose of a large majority of embryo banking cycles is short- rather than long-term banking (with the latter usually reserved for fertility preservation).

We reported in 2013, that some ART centers which performed a disproportionally large numbers of banking cycles, especially in poor-prognosis patients, reported inflated success rates reflecting preferentially selected more favorable-prognosis patients. [5] Concomitantly, we showed that the centers which reported inflated ART outcomes rapidly increased their number of ART cycles, and effectively gained market share in comparison to centers that did not follow such practices. As a result, we suggested that reporting loopholes such as this, could incentivize centers to divert especially poorer prognosis patients into banking cycles thus, enabling elimination of poor prognosis patients from statistical outcome considerations. [6] Recognition of such incentives by public ART reporting systems was especially timely since these systems were being proposed as a model for public outcome reporting by other medical and surgical specialties. [7] Our findings have been confirmed by other investigators, who found growing use of embryo banking for young poor prognosis patients leading to increasing inflation of pregnancy rates in recent years. [8]

Since our publication, the CDC and Society for Assisted Reproductive Technology (SART) have acknowledged shortcomings in the current national ART reporting systems and announced changes. [9] [10,11] Although SART is in the process of implementing these changes its most recent annual report and that of the CDC continue to embody distortions inherent to the presently utilized system.

SART website recently added a disclaimer stating: “*The data presented in this report should not be used for comparing clinics*. *Clinics may have differences in patient selection*, *treatment approaches*, *and cycle reporting practices which may inflate or lower pregnancy rates relative to another clinic”*. [12] However, neither SART nor CDC have to date acknowledged that current reports may potentially inflate overall U.S. national ART success rates.

Against this backdrop, we undertook an analysis of the degree to which exclusion of embryo banking cycles inflates national ART outcomes. As this study will demonstrate, US national ART live birth rates are significantly exaggerated, especially among older women.

## Methods

Recently made available raw data (S1 Data), [13] upon which the CDC’s 2013 Fertility Clinic Success Rates Report is based [14], were utilized in this study.

Under legal mandate, 2013 source data were self-reported to the CDC by 467 U.S. based fertility clinics. These clinics collectively performed 121,351 fresh non-donor oocyte ART cycles and 46,779 thawed non-donor oocyte ART cycles (S1 Table). Past annual validation via select on-site visits including chart reviews, suggested low discrepancy rates (<5%) for most variables. [15] Since the data are publicly available online, but cannot be utilized to identify individual patients, our study received expedited IRB approval and waiver of the need for informed consent.

To investigate the impact of embryo banking cycles on national ART outcomes, we recalculated ART live birth rates, as intended by the FCSRCA, based on number of ovarian stimulation procedures attempted. The number of ovarian stimulation procedures initiated is the number of total initiated fresh ART cycles, calculated as the number of initiated fresh ART cycles plus banked ART cycles. Total live birth rates achieved during the calendar year in fresh and thawed embryo transfer cycles were then determined per total initiated fresh ART cycles. Cycles involving use of donor oocytes were excluded.

Inflation of reported fresh ART cycle live birth rates in 2013, caused by exclusion of embryo banking cycles, was assessed for all age groups of infertile women as the difference between fresh cycle live births per number of initiated fresh cycles (excluding embryo banking cycles), as typically reported by the CDC, and fresh cycle live births per total initiated fresh ART cycles (including embryo banking cycles). Live birth rates in clinics performing more and less than the national average of embryo banking cycles were compared.

Currently published CDC data do not permit similar calculation to be performed for thawed cycles since it is impossible to determine whether thawed embryos are the product of embryo banking, represent excessive embryos from a regular fresh cycle or how many fresh cycles produced a specific cohort of thawed embryos.

All statistical analyses were performed by the center’s senior statistician (S.K.D.), using SAS version 9.4 software. Live birth rates were compared using a chi-square test, P <0.05 was considered statistically significant.

## Results

### Embryo Banking

**Fig 1:**
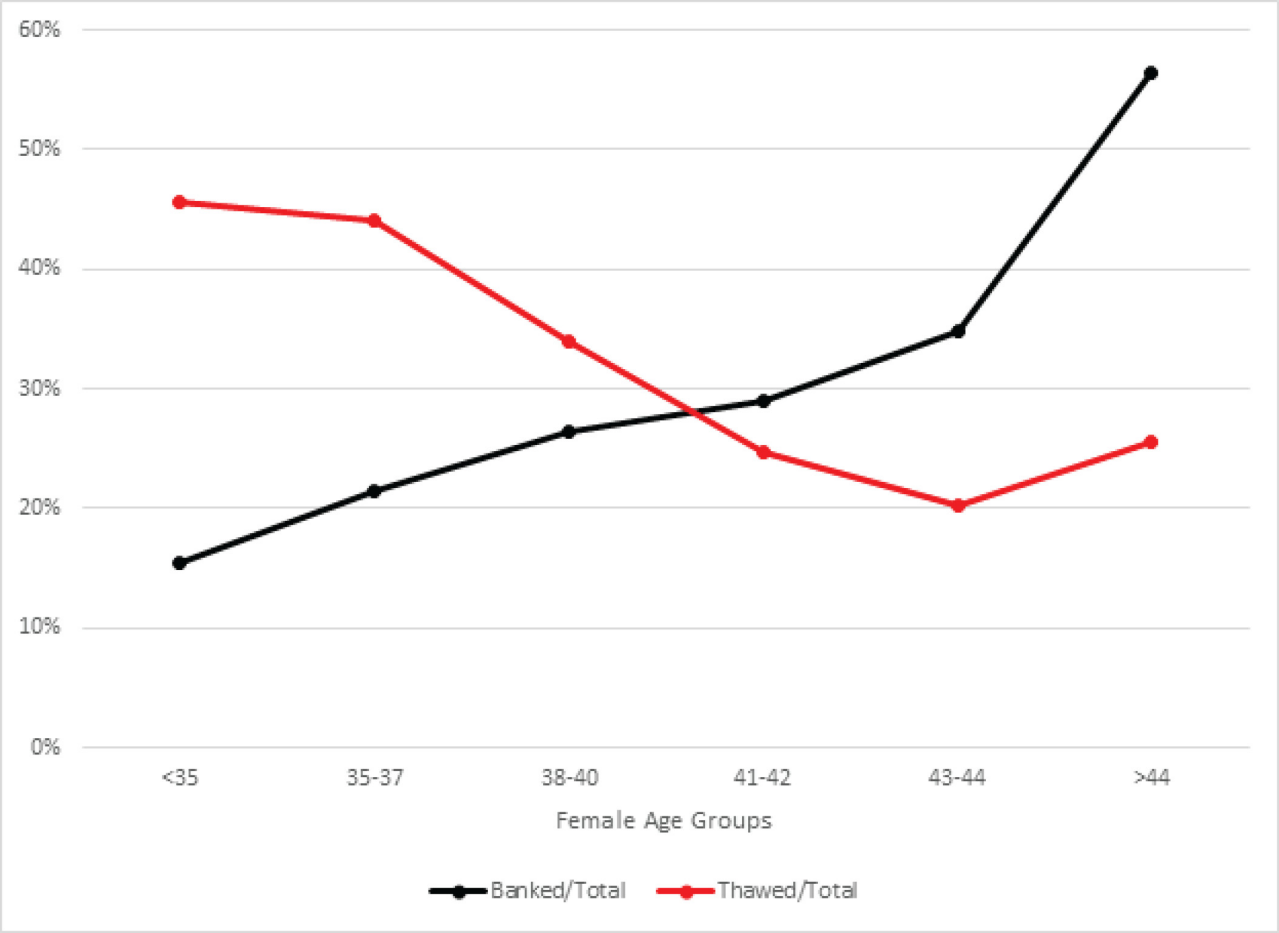
Percentage of banked and thawed cycles per total initiated fresh ART cycles in 2013.

In the year 2013, embryo banking was utilized in 27,564 out of 121,351 (22.7%) fresh non-donor ART cycles. Embryo banking cycles were more frequently performed with advancing female age, increasing from 15.5% in women <35 years to 56.5% in women >44 years old (Fig 1). Concomitantly, the proportion of thawed cycles per total initiated fresh ART cycle decreased with advancing female age from 45.6% in women <35 years to a low of 20.3% among women 43–44 years of age (P < 0.0001; Fig 1).

### ART Live Birth Rates

**Fig 2:**
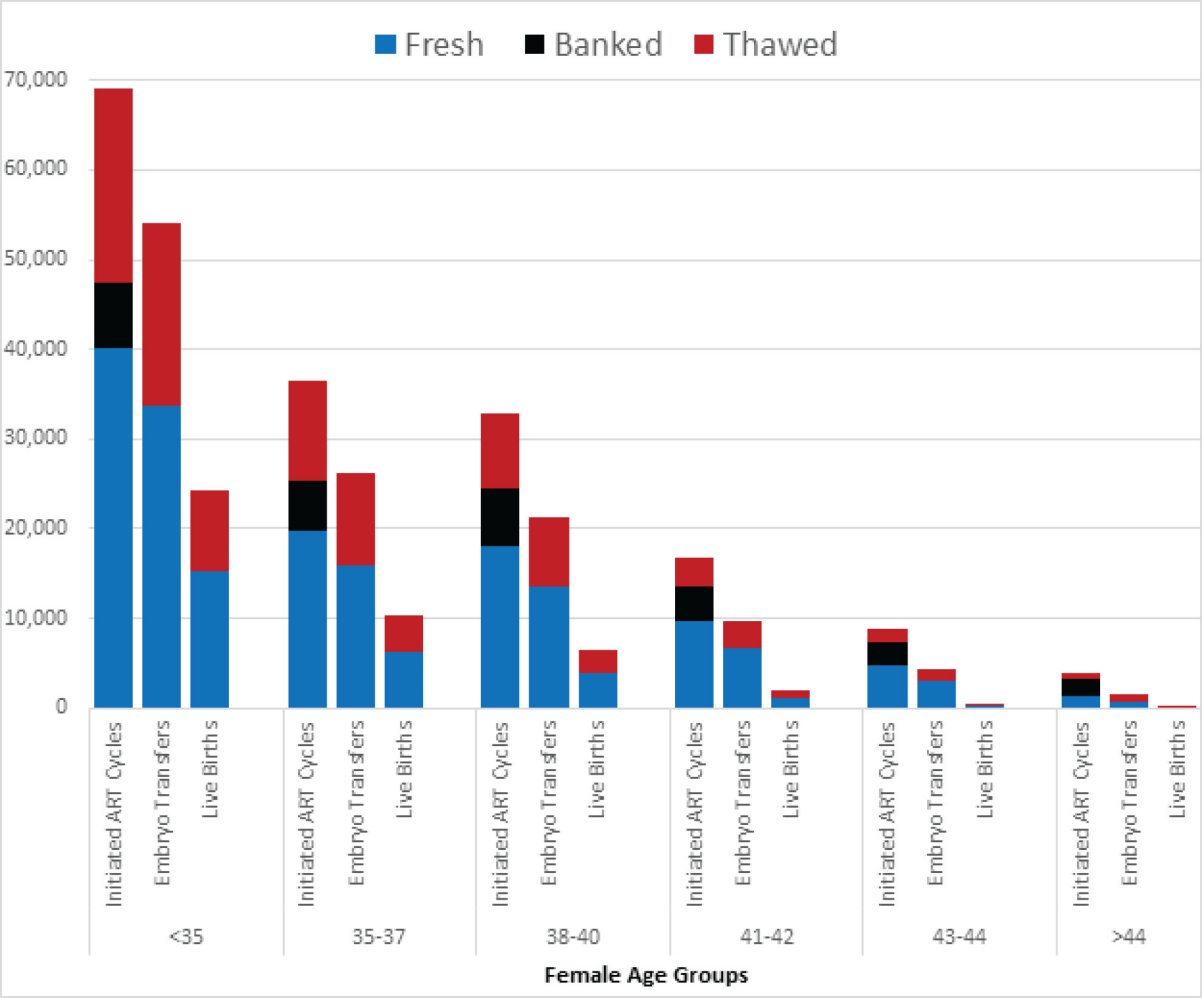
Total number of initiated ART cycles, embryos transfers and live births for each age group in 2013.

In 2013, a total of 121,351 fresh, non-donor ART cycles resulted in 44,645 live births (36.8%). Total annual live births rates (including both fresh and thawed cycles) per initiated fresh ART cycle declined with female age from 52.7% in women <35 years to only 4.0% in women >44 years (Table 1). Table 2 and Fig 2 provide additional details about how live births were derived from fresh or thawed cycles for each age group.

**Table 1:** 2013 Live Births by Age Group.

Age group	<35	35–37	38–40	41–42	43–44	>44
Live Births–Fresh Cycles	15983	6265	3819	1064	252	22
Live Births–Thawed Cycles	9002	4159	2753	910	312	104
Total Live Births	24985	10424	6572	1974	564	126
Total Live Births/ Total initiated fresh ART cycles, %	**52.7**	**41.2**	**26.8**	**14.6**	**7.6**	**4.0**

**Table 2:** Inflation of 2013 fresh ART cycle live birth rates.

Age group	<35	35–37	38–40	41–42	43–44	>44
Fresh Live Births/Fresh cycle,%	39.9	31.6	21.1	11.1	5.2	1.6
Fresh Live Births/Total initiated fresh ART cycles,%	33.7	24.8	15.6	7.9	3.4	0.7
Difference	6.2	6.8	5.6	3.2	1.8	0.9
Inflation in fresh ART cycle live birth rates *, %	**15.5**	**21.5**	**26.4**	**29.0**	**34.8**	**56.3**
Thawed Live Birth/Thawed Transfer, %	44.1	40.1	35.7	30.3	23.5	14.2
Thawed Live Births/Thawed cycle, %	41.6	37.3	33.0	27.2	20.8	12.8

* Inflation % is calculated as [(Difference)/(Fresh Live Births/Fresh cycle)] x 100

### Inflation of Fresh ART Cycle Live Birth Rates by Exclusion of Embryo Banking

The inflation in live birth rates in fresh ART cycles also increased with advancing female age and with utilization of embryo banking (Table 2). It reached a maximum in women at ages >44 years. Whereas the CDC reported a live birth rate of 1.6%, our recalculated live birth rate per total initiated fresh ART cycles was only 0.7%, reflecting a 56.3% inflation of live birth rate in this age group. Further pointing out the degree of inflation, live birth rates in thawed embryo transfer cycles for women >44 years were reported by the CDC at a very improbable 14.2% rate. As explained above, thawed cycle rates in our opinion cannot be accurately determined from currently available CDC data.

When embryo banking cycles are excluded from outcome calculations, fertility clinics performing more than the national mean of embryo banking cycles (22.7%) demonstrated similar fresh cycle live birth rates in reference to number of initiated fresh cycles and significantly higher thawed cycle live birth rates per number of initiated thawed cycles than the majority of clinics performing fewer embryo banking cycles (Table 3). However, Table 3 also demonstrates that when embryo banking cycles are accounted for, clinics performing more than the national average of embryo banking cycles actually achieved significantly lower total live births rates per initiated fresh ART cycle (absolute difference 6.0%, P<0.0001) than centers performing fewer banking cycles.

**Table 3:** 2013 Live Birth for US Fertility Clinics based on Proportion of Embryo Banking Cycles.

Embryo Banking Cycles *, %	≤22.7%	>22.7%	p-value
Number of Fertility Clinics	357	110	
Fresh Live Births/Fresh cycle,%	22125/75281 (29.4%)	5281/18506 (28.5%)	0.0225
Thawed Live Births/Thawed cycle, %	9230/27627 (33.4%)	8009/19152 (41.8%)	<0.0001
Total Live Births/ Total initiated fresh ART cycles, %	31355/80872 (38.8%)	13290/40479 (32.8%)	<0.0001

* In 2013 22.7% all U.S. fresh non-donor ART cycles were categorized as embryo banking

## Discussion

Embryo banking accounts for a rapidly growing proportion of fresh non-donor ART cycles conducted in the U.S., representing nearly a quarter of all such cycles in 2013. Though it has been known for decades that the success of cryopreservation of embryos produced with autologous oocytes declines with advancing female age, [16,17] our investigation demonstrates that, counterintuitively, embryo freezing is utilized with increasing frequency in older women. Moreover, this increased utilization with advancing female age is disconcertingly paralleled by evidence indicating that banked embryos are less likely to be used for embryo transfer with advancing age, as shown by the declining proportion of thawed cycles in older patients. Women of advanced reproductive age are, of course, the most rapidly growing population pursuing ART. [15]

Possible explanations for the observed national ART practice pattern include fewer embryos available for cryopreservation in older women, multiple embryo banking cycles to accumulate embryos for a subsequent single thaw cycle, and lower likelihood of having genetically normal embryos available following PGS in older women. At present time the CDC does not track indications for embryo banking. However, resent report suggest that the vast majority of embryo banking cycles are for purpose of short term embryo banking rather than genuine long term fertility preservation [9–11].

While embryo banking has been suggested to improve the efficacy of IVF in very young women with high response to ovarian stimulation, [3,18] appropriate prospective studies in support of this concept in more typical infertile women, who tend to be older and have average or poor response to ovarian stimulation (and, therefore average or poor ART prognoses) are lacking. Similarly, utilization of PGS, which is often accompanied by embryo banking, appears without appropriate supportive evidence preferentially directed at older women. [19,20]

Exclusion of embryo banking cycles from outcome calculations results in inflated pregnancy success rates in both fresh and thawed cycles. [5] We here demonstrate that live birth rates reported by the CDC are especially inflated in older age groups, and particularly in thawed cycles. Currently reported CDC outcomes, therefore, are misleading to the public and, in addition, are biologically implausible. For example, live birth rates per fresh ART cycle in women >44 years of 1.6%, as reported by the CDC, while already inflated by 56.3%, simply do not make sense in association with thawed live birth rates of 14.2% per transfer (of fewer embryos) in the same age group.

In reality, both figures are, likely, highly inflated, since less than half of started cycles in this age group could have reached a fresh embryo transfer, and an even smaller proportion ultimately, reach embryo transfer in a thaw cycle. Since embryo cryopreservation and thawing diminishes pregnancy and live birth chances, [16,17] lower rather than higher pregnancy rates would be expected in thawed than fresh cycles. Even assuming statistical compensation from embryo accumulation, a thawed live birth rate of 14.2% per transfer in women >44 years is biologically extremely unlikely.

We conclude that discrepancies between ART outcomes calculated and reported by the CDC, and those obtained from our recalculation of 2013 CDC data, are based on elimination of large proportions of poor prognosis patients and their embryos from statistical consideration by never allowing such patients to reach embryo transfer. Not surprisingly, this picture is particularly obvious in older women, who by definition are poorer prognosis patients than younger women. Fertility clinics performing a large number of embryo banking cycles appear to benefit in ART reports especially in thawed cycles, while actually achieving significantly lower total live birth rates per initiated ART cycle. Therefore, the current system not only misleads the public but actually incentivizes embryo banking which may lower chances for some infertile women to build a family.

CDC reports can only be improved by simplifying reported data to the most basic outcome parameter, originally intended by the Congressional national reporting mandate,—the cumulative annual chance of live birth per “number of ovarian stimulation procedures attempted.” [1,2] Therefore, the numerator should reflect the total annual number of live births from all fresh and thawed cycles. The denominator should reflect the number of fresh ART cycle starts, without exclusion for embryo banking. We have demonstrated this simple calculation here in Table 1. The main limitation of such an approach is that it may underestimate the “future” live birth from genuine long term fertility preservation cycles where a thaw may only happen in a few years. However, this is offset by including among 2013 thaw cycles, genuine fertility preservation cases that took place in prior years.

Other intermediate metrics should be deemphasized because they create confusion for the public as well as for health care providers. Such a simplified reporting paradigm would allow patients to understand chances of live birth for any given age group. A more sophisticated reporting system, accounting for additional patient characteristics and neonatal outcomes, can then be built upon such a basic reporting system.

## Supporting Information

S1 Data CDC 2013 clinic tables dataset. (XLS)

S1 Table 2013 CDC reported ART cycles by age group. (DOCX)
