# Supplementary material for: Effect of Embryo Banking on U.S. National Assisted Reproductive Technology Live Birth Rates
Source: PLoS One. 2016 May 9;11(5):e0154620. doi: 10.1371/journal.pone.0154620 (PMC4861597; doi:10.1371/journal.pone.0154620)
Supplement: S1 Table — (DOCX) [file pone.0154620.s002.docx]

**Supplemental Table 1. 2013 CDC reported ART cycles by age group**

| **Age group** | **<35** | **35-37** | **38-40** | **41-42** | **43-44** | **>44** |
| --- | --- | --- | --- | --- | --- | --- |
| Fresh Cycles | 40083 | 19853 | 18061 | 9588 | 4823 | 1379 |
| Average number of fresh embryos transferred | 1.8 | 2.0 | 2.3 | 2.7 | 2.9 | 2.5 |
| Banked Cycles | 7350 | 5437 | 6493 | 3918 | 2578 | 1788 |
| Total initiated fresh ART cycles  (Fresh + Banked Cycles) | 47433 | 25290 | 24554 | 13506 | 7401 | 3167 |
| Thawed Cycles | 21627 | 11140 | 8354 | 3344 | 1503 | 811 |
| Thawed Transfers | 20423 | 10374 | 7713 | 3005 | 1329 | 732 |
| Average number of thawed embryos transferred | 1.7 | 1.7 | 1.7 | 1.8 | 2.0 | 1.9 |
